# Switchable Multiple Spin States in the Kondo description of Doped Molecular Magnets

**DOI:** 10.1038/srep42255

**Published:** 2017-02-08

**Authors:** Rajyavardhan Ray, Sanjeev Kumar

**Affiliations:** 1 IFW Dresden, Helmholtzstr. 20, D-01069 Dresden, Germany; 2 Dresden Center for Computational Material Science, TU Dresden, 01062 Dresden, Germany; 3 Indian Institute of Science Education and Research (IISER) Mohali, Sec-81, Knowledge City, PO Manauli, Punjab, 140306, India

## Abstract

We show that introducing electrons in magnetic clusters and molecular magnets lead to rich phase diagrams with a variety of low-spin and high-spin states allowing for multiple switchability. The analysis is carried out for a quantum spin-fermion model using the exact diagonalization, and the cluster mean-field approach. The model is relevant for a number of molecular magnets with triangular motifs consisting of transition metal ions such as Cr, Cu and V. Re-entrant spin-state behavior and chirality on-off transitions exist over a wide parameter regime. A subtle competition among geometrical frustration effects, electron itinerancy, and Kondo coupling at the molecular level is highlighted. Our results demonstrate that electron doping provides a viable mean to tame the magnetic properties of molecular magnets towards potential technological applications.

Molecular Magnets (MMs) comprising of exchange-coupled cluster of transition metal ions have emerged as potential candidates for technological applications in recent years^1^,^2^,^3^. The switchability of spin states at the molecular level is central to applications in molecular spintronics, high-density magnetic data storage, and quantum computing^1^,^4^,^5^. Indeed, spin crossover (SCO) between low-spin (LS) and high-spin (HS) states has been studied in a variety of molecular complexes as a response to temperature or light^3^,^6^,^7^,^8^,^9^,^10^,^11^. Charge transfer induced SCO, either by changing the valency of metal cations or photo-induced trapping of excited spin-state, has been noted in some compounds, such as TTF-TCNQ^12^, FePc/O − Cu(110)^13^, Co-Fe Prussian blue analogues^14^ and Fe-Nb based materials^15^.

Recently, it has also become possible to control magnetism in some molecular clusters via electron doping. For example, control of intra-molecular spin alignment by charge doping in an organic molecule thiantrene bis (nitronyl nitroxide)^16^. It is also known that alkaline metal doped Copper phthalocyanine (A_*x*_CuPc) supports ferromagnetism at high temperature due to *π*–electrons^17^. Furthermore, in a three-terminal transport measurement in single-molecule magnetss (SMMs), electron transport and inelastic cotunneling effects have been observed^18^,^19^. These have been ascribed to molecular magnetic state and magnetic quantum tunneling due to Kondo interaction at the electrodes^20^,^21^,^22^.

MMs with geometrically frustrated motifs, such as triangular, tetrahedral, etc., present interesting possibilities due to ground state degeneracy. For example, it was shown by Georgeot and Mila that chirality in a triangular antiferromagnetic cluster can be controlled by external electric field and can be used as a qubit^5^. MMs with triangular or tetrahedral motifs exist with a variety of magnetic ions, such as Cr_3_^23^, many Cu_3_ complexes^24^,^25^,^26^, Mn_3_^27^, Cu_3_O^28^, V_3_^29^,^30^, Co_4_^31^, Mn_4_^32^, Ni_4_^31^, and Dy_4_^33^. However, the effect of charge doping in MMs has remained largely unexplored^34^.

A complete understanding of the doped MMs is a complex problem that requires an accurate treatment of charge, spin and vibronic degrees of freedom. As a starting point for the exploration of the possible interesting effects in doped MMs, we consider a model describing the charge and spin interactions. The vibronic coupling can affect the ground state as well as finite temperature behavior of MMs as it provides a spin decoherence pathway in some MMs^25^,^35^. However, such effects can be minimized by using chemical and physical tuning^36^. Moreover, to the best of our knowledge, a theoretical investigation of the interplay of geometrical frustration and Kondo interaction at a molecular level has never been carried out.

In this article, as a proof of concept, we show that within the Kondo description, electron-doped triangular MMs exhibit multiple low- and high-spin states. The system can be switched between different spin-states either by applying an external magnetic field or by tuning the strength of exchange parameters. The results are obtained for a quantum spin-fermion model. The case of an isolated cluster, relevant for SMMs, is analyzed using exact diagonalization. The effect of inter-cluster coupling, relevant for MMs, is included via cluster mean-field approach. The key features of our model for doped MMs, multiple spin-states, the re-entrant spin-state crossovers and chirality switching are obtained in both the approaches.

## Results and Discussions

### Doped single-molecule magnets

We begin with a model for a triangular SMM cluster doped with a single electron. The corresponding spin-fermion Hamiltonian is given by, *H* = *H*_S_ + *H*_f_ + *H*_int_, where,


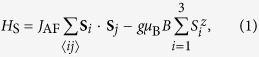



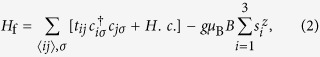



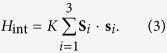


*J*_AF_ ≥ 0 is the antiferromagnetic exchange coupling between localized core spins **S**_*i*_ and *c*_*iσ*_ (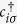) is the fermion annihilation (creation) operator at site *i* with spin *σ* = ↑, ↓. The hopping parameters in the presence of external magnetic field are given by, 
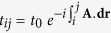
, where *t*_0_ is the bare hopping amplitude and **A** is the magnetic vector potential. The second terms in Eqs ((1)) & ((2)) represent the Zeeman coupling of the external magnetic field to the localized and itinerant spins, respectively. *K* denotes the strength of on-site Kondo coupling between localized spins **S**_*i*_ and itinerant spins **s**_*i*_. Both ferromagnetic, *K*_F_, and antiferromagnetic, *K*_AF_ cases will be considered. In the following, we set *g* = 2, and *t*_0_ = 1 serves as the reference energy scale.

The lattice version of the above model has been intensively studied in the context of manganites and itinerant frustrated magnets^37^,^38^,^39^,^40^. However, the localized spins are treated classically in most such studies. The cluster version of this model is experimentally relevant for SMMs, quantum dots, and nanoscale devices. Experimentally, quantum tunneling of magnetic moments in single MMs, such as, Mn_12_ and Fe_8_, has also been reported^18^,^19^, suggesting the significance of quantum effects. Therefore, in our study it is of paramount importance to treat the localized spins as operators rather than classical spins.

In the context of bulk transition metal systems, such as manganites, the Kondo description is befitting due to mixed valency of the transtion metals. In such a scenario, the system lowers the ground state energy via delocalization of the additional electron/hole. In the process, an effective coupling between localized magnetic moments is mediated via the itinerant electrons^41^,^42^. The competition between the primary superexchange coupling and this additional induced coupling is known to generate non-trivial magnetic phases in lattice models^39^. Simiar situation may arise in the MMs as well, and is a problem of significant practical relevance. In fact, mixed valency as been noted in some Mn-based di-nuclear and polynuclear transition metal compounds^32^. Furthermore, the above Hamiltonian also finds relevance in a host of di-nuclear transition metal clusters^43^,^44^,^45^,^46^,^47^,^48^. Additionally, a Kondo description is also known to be relevant when considering the interaction of MMs with metal contacts and/or substrate^20^,^21^,^22^,^49^.

We employ the exact diagonalization method to study the above Hamiltonian. The dimension of the Hilbert space turns out to be 2*Nm*(2*S* + 1)^*N*^ = 48 for one itinerant electron (*m* = 1) and three *S* = 1/2 cluster spins (*N* = 3). The access to exact eigenstates and eigenvalues allows us to compute all spin-spin and spin-fermion correlation functions exactly. The ground states are characterized by, (i) the spin-spin correlations, *D*_*ij*_, (ii) the spin-fermion correlations, *F*_*ij*_, and (iii) the total spin, *S*_tot_.

**Figure 1:**
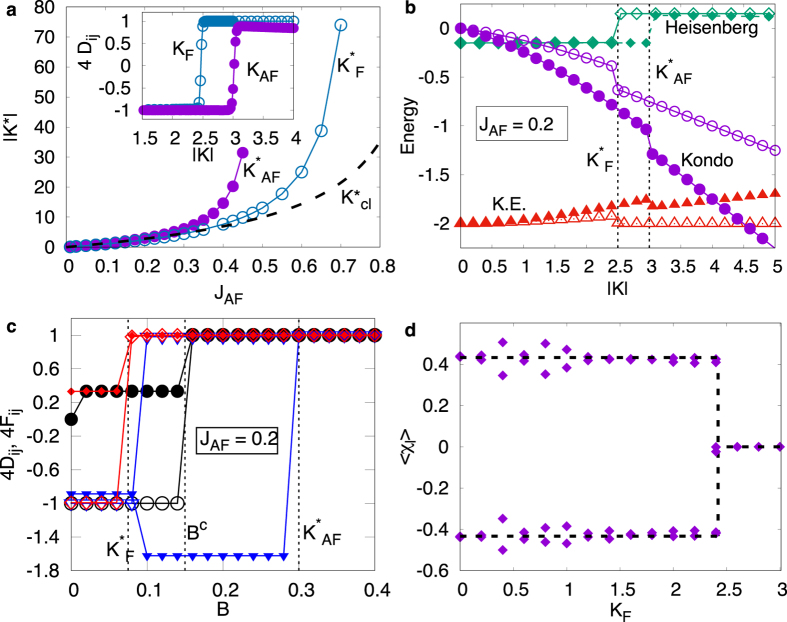
(**a**) *J*_AF_ dependence of the critical value K^*^ of the Kondo coupling for crossover from a low-spin to a high-spin state. The crossover is characterized by the spin-spin correlations shown for both FM and AFM Kondo couplings in the inset. (**b**) The ground state energy contributions of the kinetic, the Heisenberg and the Kondo terms as a function of *K*_F_ (open symbols) and *K*_AF_ (filled symbols). (**c**) Spin-spin (open symbols) and spin-fermion (filled symbols) correlations as a function of external magnetic field for *J*_AF_ = 0.2 for different values of *K: K* = 0 (circle), *K*_F_ = 1.0 (diamond) and *K*_AF_ = 1.0 (triangle). The dashed lines mark the values of *B*^c^, 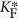 and 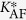. (**d**) Chirality of each of the eigenvector forming the degenerate ground state manifold as a function of *K*_F_.

We start our discussion with the observation that, for *K* ≠ 0, Kondo spin fluctuation (KSF) processes are possible, *i.e.* the core spins can flip at the expense of the fermionic spin and vice versa. We also note that for *K* < 0 (*K* > 0) the core spins and the electronic spins tend to align parallel (anti-parallel) to each other. We label these couplings as *K*_F_ and *K*_AF_, respectively. The case of an undoped triangular cluster has been carefully analyzed before, and as one expects for AFM interactions the spin-spin correlations are negative for all spin pairs^5^. We find that a single electron introduced in the cluster leads to an abrupt switching of the sign of *D*_*ij*_ beyond a critical value of Kondo coupling (see inset in Fig. 1(a)). The abrupt change can be explained in terms of spin-state switching of the doped cluster. The critical value K^*^ required for switching depends on not only the value of the AFM exchange, but also the sign of the Kondo coupling, as shown in Fig. 1(a). Assuming the localized spins to be classical, the switching can be understood as an outcome of the competition between the AFM exchange, *J*_AF_, and the FM exchange arising via double-exchange (DE) mechanism (see Methods).

In order to clearly understand the origin of the spin-state switching driven by Kondo coupling, we compute the contribution of each term in the Hamiltonian to the total ground state energy. Upon increasing Kondo coupling strength, the kinetic energy increases, the contribution of the coupling term decreases and the contribution of the Heisenberg term remains constant (see Fig. 1(b)). At K^*^ both the kinetic and the Kondo contributions to total energy are lowered at the cost of an increase in the Heisenberg contribution, leading to an overall lowering of energy. Interestingly, for large *K* the Kondo contribution varies linearly with a slope of −*K*_F_/4 and −*K*_AF_/2 for the two possible signs of the Kondo coupling. This dependence can be explained in terms of the KSF processes which are dominant in the case of AFM Kondo coupling.

We show that the spin-state switching can also be driven by a magnetic field. This is inferred from the calculations of spin-spin and spin-fermion correlations shown in Fig. 1(c). The spin-fermion correlations are vital in identifying the key differences between states that possess an identical value of *S*_tot_. This will be discussed later. The spin chirality is defined as *χ*_*l*_ = 〈**S**_*i*_ ⋅ (**S**_*j*_ × **S**_*k*_)〉_*l*_, where 〈…〉_*l*_ denotes the quantum expectation value in a state *l*. We find that 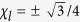 for each of the degenerate ground state for 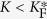, and becomes identically zero for 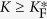 (see Fig. 1(d)). This is particularly relevant in view of the recent proposal of utilizing chirality of a triangular MM as a qubit in quantum computations^5^. The electron doping can provide another knob for switching on and off the chirality of the system.

**Figure 2:**
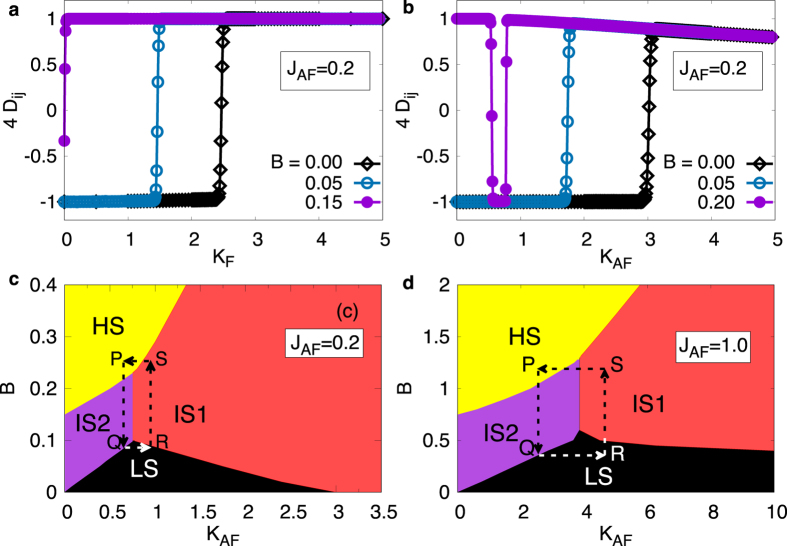
(**a**,**b**) Spin-spin correlations as a function of (**a**) *K*_F_ and (**b**) *K*_AF_ for different values of the external magnetic field. (**c**,**d**) The phase diagram showing different spin-states of the doped triangular cluster in the *B* − *K*_AF_ plane for (**c**) *J*_AF_ = 0.2 and (**d**) *J*_AF_ = 1.0. HS and LS, respectively, correspond to High-Spin and Low-Spin state while IS1 and IS2 are Intermediate Spin states.

We have already shown that external magnetic field can serve as a control parameter to switch between different spin-states. Indeed, for an undoped cluster applying large external magnetic field, *B*, leads to a high-spin ground state, HS, characterized by **S**_tot_ = 3/2 and *D*_*ij*_ = 1/4 = *F*_*ij*_ (∀*ij*). As discussed earlier, the low- to high-spin state crossover can also be obtained by tuning the Kondo coupling. For the FM Kondo coupling *K*_F_, the influence of magnetic field is to lower the critical value 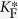, as shown in Fig. 2(a). Hence, both *K*_F_ and *B* work together to destabilize the low-spin state. Eventually, beyond a critical value of *B*, which is *B*^c^ = 3*J*_AF_/4 for an undoped MM cluster (*K* = 0), the low-spin state ceases to exist.

The situation for *K*_AF_ is strikingly different. Figure 2(b) shows the spin-spin correlations as a function of *K*_AF_ for different values of *B*. In this case, the Zeeman coupling term also competes with the KSF processes leading to an interesting magnetic field dependence. At small values of *B*, the 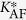 for crossover from low-spin to high-spin state decreases upon increasing *B*, similar to the *K*_F_ case. However, for *B* ≳ *B*^c^, the ground state shows an unexpected re-entrant double-switching behavior (see Fig. 2(b)). The understanding of this, as discussed below, highlights the subtle competition of different energy terms that is at play in this simple model. In the weak Kondo coupling limit, the Zeeman term wins over the Kondo and *J*_AF_ terms, leading to fully polarized HS state. Upon increasing *K*_AF_, the energy gain from the Zeeman term is exactly compensated by the contributions from the Kondo term and the core spin texture switches to AF. As *K*_*AF*_ is further increased, the ground state switches back to a high-spin state where the magnetic field can overcome the *J*_AF_ term but the KSF processes are dominant over the Zeeman term, resulting in IS1 state.

**Figure 3:**
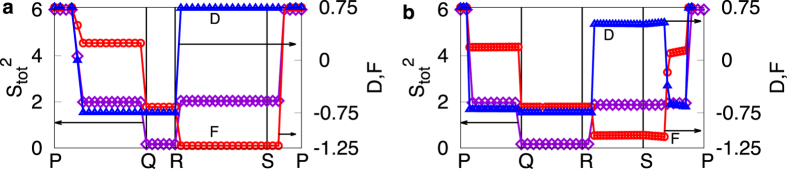
Change in total spin (violet diamonds), spin-spin correlations, *D* = ∑_*ij*_*D*_*ij*_ (blue triangles), and spin-fermion correlations, *F* = ∑_*ij*_*F*_*ij*_ (red circles) for (**a**) *J*_AF_ = 0.2 and (**b**) *J*_AF_ = 1.0, upon varying parameters along the path shown in Fig. 2(c,d), respectively.

For *B* ≪ *B*^c^, while the core spin texture is AF, with increasing values of *K*_AF_, the spin-fermion correlation switches from FM to AF due to energy gain from the Kondo term. With further increase in the Kondo coupling, the core spin texture switches from AF to FM while the spin-fermion correlations remain AF, leading to yet another double-switch behavior between the IS2, LS and IS1 states. We summarize these findings as a phase diagram in Fig. 2(c–d). The important aspect is the possibility of multiple switchability by either the external magnetic field or the strength of Kondo coupling. We illustrate this by plotting the spin-spin and spin-fermion correlations together with the total spin along closed-loop paths indicated in the phase diagrams (Fig. 3(a,b)).

An accurate modelling of a real system comes at the cost of simultaneously introducing a large number of competing effects and the corresponding parameters. Here, we have tried to keep the number of parameters to a minimum in order to focus on the elementary effects. As stated earlier, the effect of spin-lattice coupling can be very important for certain SMMs. One possible effect of such a coupling is to lower the symmetry of the cluster, for example in the present context, from equilateral triangle to isosceles triangle. This will lead to an inequivalence of the Heisenberg couplings and the electronic tunneling amplitudes. It is worth noting that most of the tri-nuclear (3-site) MM clusters are found to be in a lower symmetry group than that of an equilateral triangular geometry considered in this work. We verify that the effects in terms of spin-state switching are robust against such symmetry lowering induced by additional interactions that may be present in SMMs (see Supplementary Material).

### Doped Molecular Magnets

The magnetic clusters in MMs are not completely isolated^50^. However, the inter-cluster coupling is typically weak and therefore the analysis of an isolated cluster provides insights into the behavior of the MMs^18^,^19^. Nevertheless, it is important to verify the stability of various spin-states upon including the effect of inter-cluster coupling. We now present this analysis within a cluster mean-field (CMF) approach^51^,^52^. We assume that the doped cluster is surrounded, on the average, by *z* other clusters of which a fraction *p* are doped. The inter-cluster (IC) interaction is approximated by the exchange interaction between total spins of these clusters, leading to the extended Hamiltonian *H*′ = *H* + *H*_*IC*_, with,


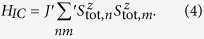


In the above, *J*′ is the exchange coupling between two clusters and 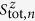 is the *z* component of the total spin on *n*^*th*^ cluster. The primed sum refers to summation over all *z* neighbors of a given cluster. The ground state is obtained variationally including symmetry-broken states as well (see Methods).

**Figure 4:**
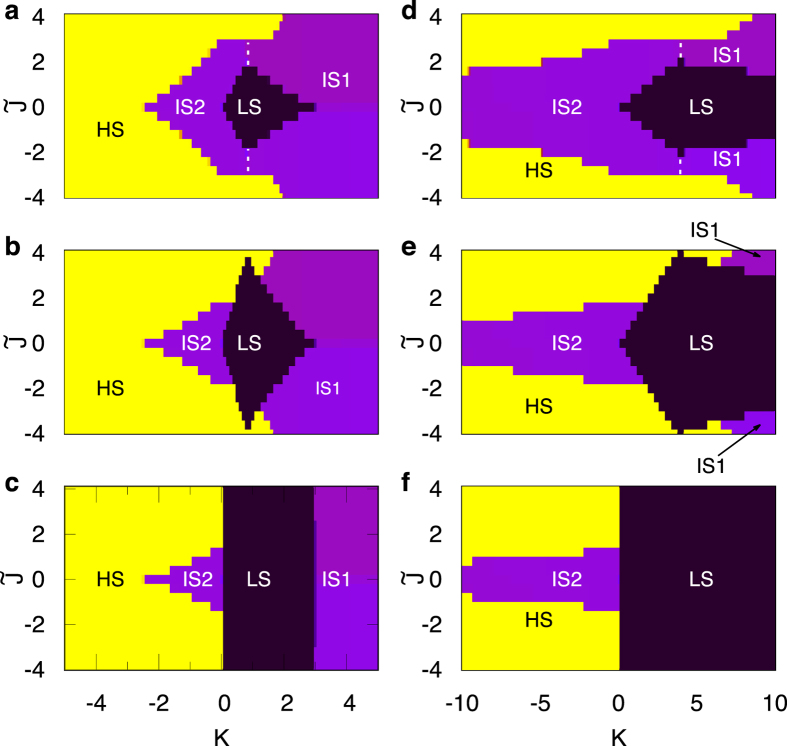
The phase diagram for spin-switchability in doped triangular MMs as a function of normalized exchange coupling 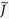 and *K* for *J*_AF_ = 0.2 (**a**–**c**) and *J*_AF_ = 1.0 (**d**–**f**). The values of doping fraction used are, *p* = 0 (**a**,**d**), *p* = 1/2 (**b**,**e**) and *p* = 1 (**c**,**f**). HS and LS, respectively, correspond to High-Spin and Low-Spin state while IS1 and IS2 are Intermediate Spin states. Different colors refer to the values of 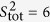 for HS (yellow), 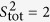 for IS1 and IS2 (violet), and 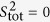 for LS (black).

It is clear that various phase boundaries will scale with the coordination number *z*, therefore we define 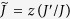 as an effective measure of the IC coupling. Figure 4 shows the complete phase diagram for different values of *J*_AF_, *K* and 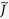, for *z* = 4 and 0 ≤ *p* ≤ 1. For 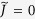, the phase boundaries are consistent with the results for SMMs discussed above. Slightly away from 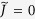, for *p* = 0 (Fig. 4(a,d)), the SMM cluster is surrounded only by undoped SMMs. Although the net magnetic moment is zero, the ground state corresponds to the symmetry-broken mean field which acts like an external magnetic field coupled to the total spin. Therefore, as for the SMMs, the phase space is divided into four (two) distinct regions for *J*_AF_ = 0.2 (*J*_AF_ = 1.0). The intra-molecular spin fluctuation processes are suppressed due to effective field arising from the neighboring clusters. Hence, the re-entrant IS2 state is destabilized in favor of HS state at the IS2-IS1 and HS-IS1 boundary, for *K*_AF_ (*K* > 0).

At finite values of *p*, the ground state of doped MMs is driven by the KSF processes. Therefore, the effective MF generated is less than that for *p* = 0. While the ground states in the DE limit (|*K*|/*t* ≫ 1) remain unchanged, the state LS (IS2) is stable over an increasingly larger (smaller) range of parameters with increasing values of *p*. Eventually, for *p* = 1 and *J*_AF_ = 0.2, there is a LS-IS1 SCO at *K*_AF_ = 3 independent of 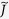. On the other hand, for *J*_AF_ = 1.0, the LS state exists over the entire range of *K*_AF_ and 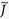 values. It is easy to see from the phase diagram that multiple SCOs can be realized by varying suitable parameters. SCO between three distinct states acts as a tri-stable switch and hold promise for technological application such as spintronics and as ternary qubits in quantum computation^53^,^54^,^55^.

Regarding the experimental accessibility of the different regions in the phase diagram, we observe that the exchange coupling in MMs is typically small, of the order of few meV^30^. However, depending on the MM, this value could vary between 0.2 me*V* for V_3_^30^ to 62 meV for Cu_3_-Pyrazolato complexes^26^. Even among the MMs involving the same transition metal, the AF exchange coupling, *J*_AF_, differs by an order of magnitude, between Scalene (*J*_AF_ ~ 2–4 meV for Cu_3_(OH))^25^ and the Cu_3_-Pyrazolato complex mentioned above, both of which involve Copper. Also, the hopping parameter could vary significantly depending on the nature of the ligands connecting the transition metal ions. Similarly, the Kondo coupling is also material specific. Therefore, different triangular MMs would belong to different regions of the phase diagram. One approach to theoretically obtain the relevant parameters for currently synthesized MMs would be to perform *ab-initio* density functional theory based calculations. The phase diagrams presented here will serve as a crucial reference for any future work in this direction.

Nevertheless, it is interesting to note that different regions of the phase diagrams could be accessed by carefully tuning the parameters. It has been proposed and reported that exchange couplings as well as the Kondo coupling strengths can be modulated by an STM set up^56^, engineering the MM-metal contact^49^, gate voltage in a transport set-up^57^, deliberate structural distortions^58^, and chemical bond manipulation^59^. With increasing handle on the parameter space, it may soon be possible to tune the MMs into desired configurations where the interesting effects discussed above could be accessed and utilized.

## Conclusions

As a simple model for charge-doped MMs, we have studied the competition between exchange interaction, Kondo coupling, and electron itinerancy in a triangular magnetic cluster. For an isolated SMM, the effect of *K*_F_ and *K*_AF_ are fundamentally different in terms of Kondo spin fluctuation processes as well as scalar spin chirality. The presence of Kondo spin fluctuation processes destabilize the LS ground state in favor of intermediate- and high-spin states. Furthermore, an external magnetic field leads to a “double-switch” behavior for *K*_AF_ allowing for a re-entrant spin-state crossover. Together with the fermionic response, this leads to a rich phase diagram allowing for multiple SCOs. The results are robust against the effects of inter-cluster interactions as analyzed within cluster mean field approach. Our results open up an exciting possibility of tuning the properties of MMs for applications in data storage and processing devices where multiple switchability at the molecular level is desired. This work should motivate further theoretical and experimental investigations on doped MMs with geometrically frustrated motifs.

## Methods

### Characteristics of Different Ground states

The different spin-states realized for the doped SMMs and MMs are characterized in terms of the spin-spin correlations, spin-fermion correlations, and total spin in the ground state, respectively, defined as:


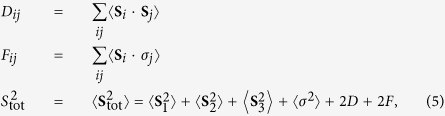


where *D* = ∑_*ij*_*D*_*ij*_, and *F* = ∑_*ij*_*F*_*ij*_. While HS corresponds to the fully polarized high-spin state with FM correlations, LS is the low-spin state corresponding to AF spin-spin and spin-fermion correlations. The ground state properties of different states realized above is presented in Table 1.

It is interesting to note the values of spin-spin and spin-fermion correlations for the IS1 state in the limiting cases. Further insights into this state can be obtained by studying the pure Kondo model, corresponding to *J*_AF_ = 0, where only HS and IS1 states are realized in the ground states. Furthermore, the ground state properties of one-electron Kondo lattice model on a triangular cluster is found to be remarkably similar to the corresponding results for a triangular lattice (see Supplementary Material for details).

### Charge-Charge Correlations

It turns out that the fermion is equi-distributed on all the sites due to degeneracy in the ground state, 〈*n*_*i*_〉 = 1/3 (for *i* = 1, 2, 3). However, differences in different ground states is also reflected in their respective charge-charge correlations, defined as:


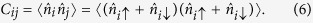


**Figure 5:**
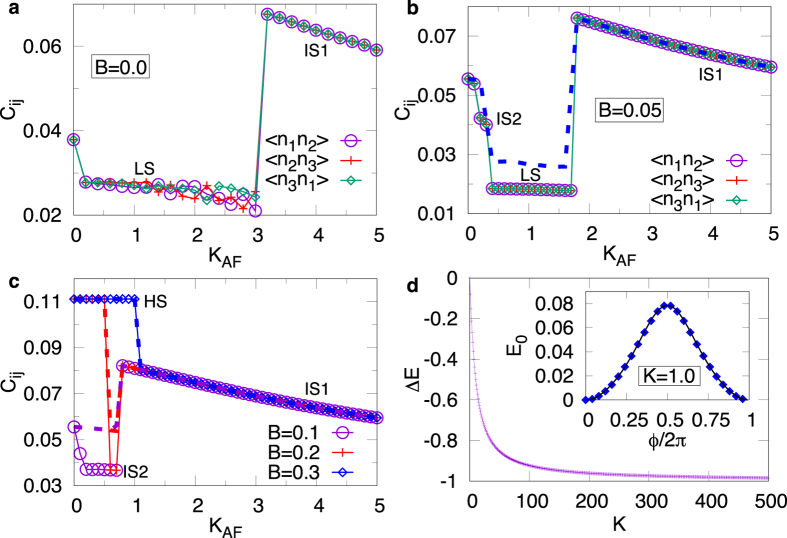
(**a**–**c**) Charge-charge correlations as a function of *K*_AF_ at *J*_AF_ = 0.2, for different values of *B*: (**a**) B = 0, (**b**) B = 0.05 and (**c**) B = 0.1, 0.2 & 0.3. All {*ij*} pair are identical in panel (**c**). The dashed lines represent the calculations without the Peierls substitution. (**d**) The change in ground state energy as one of the spins in a classical triangular ferromagnet is rotated by an angle *π*. The inset shows the change in ground state energy as *ϕ* is varied continuously from 0 to 2*π* (symbols) and a fit (line) to *f(x*) = *a* + *b* cos(*ϕ*) + *c* cos^2^(*ϕ*) + *d* cos^3^(*ϕ*) with *a* = 0.0293, *b* = −0.0345, *c* = 0.0097, and *d* = −0.0047.

The charge-charge correlations for the HS state corresponds to the non-interacting behavior while other states show deviation. Figure 5(a–c) show the behavior of charge-charge correlations at different values of *B*, for *J*_AF_ = 0.2. For *B* = 0 (panel (a)), with increasing values of *K*_AF_, the ground state switches from LS to IS1 state at 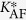. While the characteristic feature of IS1 state is same as that for *J* = 0 (see Supplementary Material for details), the charge-charge correlations for LS phase decreases continuously and shows fluctuations with increasing values of *K*_AF_, particularly near 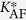.

For *B* = 0.05 (panel (b)), we see three distinct charge-charge correlation values consistent with the phase diagram discussed earlier. At finite values of *B*, the charge-charge correlations for LS phase is: 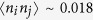 for all {*ij*} pairs. The IS2 state shows a charge correlations slightly higher than the corresponding values for LS. This is further highlighted in panel (c) which shows the charge-charge correlations at even higher values of *B*. Compared to IS2, the charge-charge correlations for IS2 state is: 〈*n*_*i*_*n*_*j*_〉 ~ 0.037 for all {*ij*} pairs. Similar behavior of charge-charge correlations is also seen for *J*_AF_ = 1.0.

We find that different spin-state switchings are driven by competition between the kinetic energy, Zeeman coupling, AF exchange interaction and Kondo induced ferromagnetic spin-spin couplings. Also, it is worth highlighting that, for the range of magnetic fields considered in this work, Peierls substitution in the kinetic energy term plays no role in the spin-state switching. For fully polarized HS state and the IS1 state, where the core spin texture is FM, this terms does not affect the charge-charge correlations as well. However, for LS and IS2 state, where the core spin texture is AF, the charge-charge correlations in the absence of Peierls term is different, as shown with dashed lines in Fig. 5(c,d).

### Kondo Interaction Induced Effective Exchange Coupling

In order to obtain a crude estimate of the effective exchange coupling induced by the Kondo interactions, we calculate the change in energy of a classical triangular ferromagnet cluster as one of the spins is rotated by *ϕ* = *π*. Figure 5(d) shows the change in energy as a function of |*K*|. The effective exchange coupling, being equal to the change in energy, is clearly ferromagnetic, and in the DE limit, is approximately 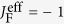. At finite values of *K*, higher order Kondo terms also contribute. We find small, but significant, contributions arising from higher order processes consisting of *t*^4^/*K*^3^ (proportional to (**S**_*i*_ ⋅ **S**_*j*_)^2^) terms. The inset shows the behavior of the ground state energy as one of the spin is rotated by an angle 0 < *ϕ* ≤ 2*π*, for |*K*| = 1 alongwith a fit as function of various powers of cos(*ϕ*).

Notwithstanding the fact that the quantum corrections have not been taken into account in the above analysis, Fig. 1(a) shows a comparison of the extracted 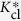 from the above analysis and K^*^ obtained from the exact diagonalization (ED) calculation. At small values of *J*_AF_, we find that the extracted value of 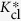 is in good agreement with the corresponding ED results. However, as *J*_AF_ increases, the quantum fluctuations lead to deviations from the classical values obtained above.

### Cluster Mean Field Approximation

For studying the interaction of a doped triangular MM with *z* surrounding MM clusters, we consider a generic situation where a fraction *p* of the neighboring MM clusters are doped. Since charge clustering will be suppressed due to repulsive Coulombic interactions, we assume that clusters are either undoped or doped with one extra electron. Within a mean-field treatment, the neighboring clusters interact only via exchange interactions between total spins, *S*_tot_, on each cluster. The resulting mean-field Hamiltonian is given by,


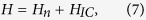


where, *H*_*n*_ is the intra-cluster Hamiltonian for cluster *C*_*n*_ and *H*_*IC*_ is the exchange interaction with neighboring clusters *C*_*m*_. For simplicity, we call these clusters *n* and *m*, respectively. The explicit forms of the *H*_*n*_ and *H*_*IC*_ are given by^51^,^52^:


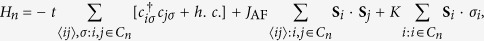


and,


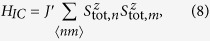


where *t, J* and *K* represent the hopping strength, exchange coupling and Kondo coupling strength for cluster *n*, and *J*′ in the exchange coupling between the neighboring clusters. *c*_*i,σ*_, **S**_*i*_ and *σ*_*i*_ are the fermion creation operator at site *i* with spin *σ* = ↑, ↓, the on-site core spin and fermionic-spin operators. 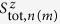 represents the *z*-component of the total spin in cluster *n (m*).

We treat the inter-cluster interaction within the Mean-Field (MF) approximation. Ignoring the higher order terms, we have:


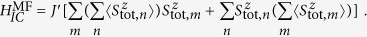


Collecting all terms for the cluster *n*, we obtain:


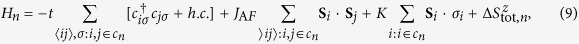


where 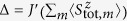 is the MF experienced by cluster *n*. In order to obtain a self-consistent solution, we start with an arbitrary value of Δ in the above Hamiltonian, diagonalize it and compute 〈**S**_tot_〉 for an undoped and a doped cluster. The value of Δ is given by:


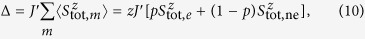


where, 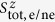 is the total spin for a doped/undoped MM cluster. The computed values of Δ is used as input in the next step. The above steps are repeated iteratively until the average difference between the Δ values of two successive iterations becomes less than 10^−5^, leading to a self-consistent MF solution. This procedure was carried out for different initial values of Δ so as to include the symmetry-broken states. The total energy of the self-consistent solutions was then minimized in order to obtain the ground states.

It should be noted that this approach is independent of the geometric arrangement of clusters and, in principle, allows for all values of cluster coordination (# of NN clusters): *z* = 1, 2, 3, 4, 5, 6, …. However, the corresponding values of *p* get restricted due to the physical constraint that the number of neighboring clusters which are doped can only be an integer. Table 2 lists the allowed *z*- and corresponding *p*-values. The values of *p* = 0 (*p* = 1) corresponds to none (all) of the neighboring cluster being doped with an extra electron, implying that there is only one doped MM cluster for *p* = 0 and *z* + 1 doped clusters for *p* = 1.

## Additional Information

**How to cite this article**: Ray, R. and Kumar, S. Switchable Multiple Spin States in the Kondo description of Doped Molecular Magnets. *Sci. Rep.* **7**, 42255; doi: 10.1038/srep42255 (2017).

**Publisher's note:** Springer Nature remains neutral with regard to jurisdictional claims in published maps and institutional affiliations.

## Supplementary Material

Supplementary Information

## Figures and Tables

**Table 1:** Characteristics of Different Ground States realized for doped SMMs and MMs.

State	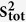	*D* = ∑_*ij*_*D*_*ij*_	*F* = ∑_*ij*_*F*_*ij*_
HS	6	3/4	3/4
IS1	2(*K*_AF_/*t* ≪ 1)	3/4	−5/4
	(*K*_AF_/*t* ≫ 1)	1/4	−3/4
IS2	2	−3/4	1/4
LS	0	−3/4	−3/4

**Table 2:** Allowed *z, p* values.

*z*	allowed *p*-values
1	0, 1
2	0, 1/2, 1
3	0, 1/3, 2/3, 1
4	0, 1/4, 2/4, 3/4, 1
5	0, 1/5, 2/5, 3/5, 4/5, 1
6	0, 1/6, 2/6, 3/6, 4/6, 5/6, 1
