# Supplementary material for: Switchable Multiple Spin States in the Kondo description of Doped Molecular Magnets
Source: Sci Rep. 2017 Feb 8;7:42255. doi: 10.1038/srep42255 (PMC5296747; doi:10.1038/srep42255)
Supplement: Supplementary Information [file srep42255-s1.pdf]

Supplementary Material for "Switchable Multiple Spin States in the Kondo description of Doped Molecular Magnets"

Rajyavardhan Ray[1,2,*] **and Sanjeev Kumar**[3,†]

[1]Institute for Theoretical Solid State Physics, IFW Dresden, PO Box 270116, D-01171 Dresden, Germany
[2]Dresden Center for Computational Material Science, TU Dresden, 01062 Dresden, Germany
[3]Indian Institute of Science Education and Research (IISER) Mohali, Sec-81, Knowledge City, PO Manauli, Punjab, India - 140306
[*]r.ray@ifw-dresden.de
[†]sanjeev@iisermohali.ac.in

ABSTRACT

In this supplement, we present the calculations for electron-doped triangular single molecule magnet for $J_{\rm AF}=0$. In particular, we calculate spin-spin correlations, spin-fermion correlations, charge-charge correlations and total spin for the cluster. Interestingly, the results for a cluster are in good agreement with the corresponding results on the lattice. We also show that the results presented in the main text can be generalized to asymmetric triangular and 4-site clusters.

One-Electron Kondo Model

The ground state for one conduction electron hopping on a lattice formed by core spins and interacting with them via Kondo interactions (Kondo lattice model) has been studied earlier[1]. In the study, hopping energy of the conduction electron and the Kondo interaction strength were the only energy scales. This corresponds to setting the exchange coupling and magnetic field equal to zero in the Hamiltonian studied in the present work (Eq. (1), (2) & (3) in the main text). The ground state of such a system is an incomplete (complete) ferromagnet (FM) with $S_{\rm tot}=(N-1)/2$ $(=(N+1)/2)$ for $K_{\rm AF}$ ($K_{\rm F}$). Here, we verify that these general results hold for a triangular SMM doped with one electron. We explicitly obtain the behavior of spin-spin, spin-fermion, and charge-charge correlations as a function of K, with emphasis on the fact that, for $K_{\rm AF}$, these quantities are significantly different in the limiting cases.

We start with the Hamiltonian,

$$H=-t\sum_{\langle ij\rangle,\sigma}(c^{\dagger}_{i\sigma}c_{j\sigma}+h.c.)+K\sum_{i=1}^{3}\mathbf{S}_i\cdot\mathbf{s}_i\ , \tag{1}$$

as obtained from Eq. (1,2, & 3) in the main text, with $B=0$, $J_{\rm AF}=0$. $\mathbf{S}_i$ ($\mathbf{s}_i$) is the on-site core spin (fermion spin) operator. For simplicity, we introduce the following notations for total spin, spin-spin and spin-fermion correlations:

$$S=\langle\sum_i\mathbf{S}_i\rangle\ ;\ \ s=\langle\sum_i\mathbf{s}_i\rangle\ ;\ \ S_{\rm tot}=\langle\mathbf{S}+\mathbf{s}\rangle\ ;\ \ D=\langle\sum_{ij}\mathbf{S}_i\cdot\mathbf{S}_j\rangle\ ;\ \ F=\langle\sum_{ij}\mathbf{S}_i\cdot\mathbf{s}_j\rangle\ , \tag{2}$$

where, $\langle...\rangle$ represents the expectation values. Therefore, $\langle\mathbf{S}^2_{\rm tot}\rangle$ is defined as:

$$S^2_{\rm tot}=\langle\mathbf{S}^2_{\rm tot}\rangle=\langle\mathbf{S}^2_1\rangle+\langle\mathbf{S}^2_2\rangle+\langle\mathbf{S}^2_3\rangle+\langle\sigma^2_2\rangle+2D+2F \tag{3}$$

The on-site electron occupation for site i and spin $\sigma=\uparrow,\downarrow$ is represented by the expectation value of the number operator, $\langle\hat{n}_{i\sigma}\rangle$, and the charge-charge correlations between sites i and j is given by:

$$C_{ij}=\langle\hat{n}_i\hat{n}_j\rangle=\langle(\hat{n}_{i\uparrow}+\hat{n}_{i\downarrow})(\hat{n}_{i\uparrow}+\hat{n}_{i\downarrow})\rangle\ . \tag{4}$$

The ground state expectation values of these operators is calculated over the energy spectrum using the partition function at very low temperatures ($k_{\rm B}T=0.001$).

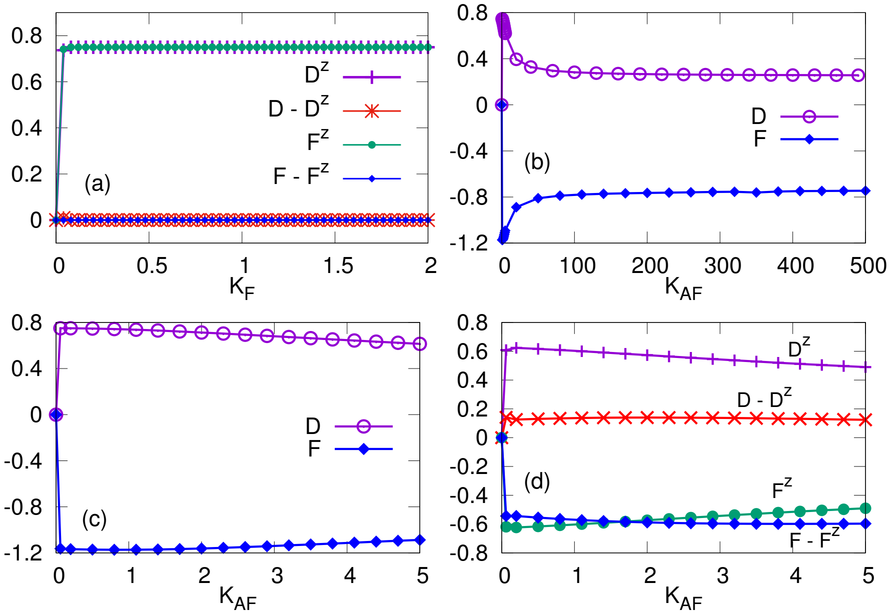

Figure 1. The spin-spin and spin-fermion correlations as a function of K for: (a) K_{F} and (b)-(d) K_{AF}. Please refer text for details.

Spin-spin and Spin-fermion Correlations

For K_{F} ($K < 0$), the ground state is a fully polarized (complete) ferromagnet. Fig. 1(a) shows the behavior of spin-spin correlations and the spin-fermion correlation as a function of Kondo coupling. We find that:

$$\begin{aligned} D &= 3/4 \,, \\ \text{and,} \quad F &= 3/4 \,, \end{aligned} \tag{5}$$

leading to

$$S_{\mathrm{tot}}^2 = 6 \,. \tag{6}$$

Also, the transverse components are identically zero, in agreement with:

$$\begin{aligned} S_{\mathrm{tot}} &= (N+1)/2 = 2 \,, \\ S^z &= S_{\mathrm{tot}} \,. \end{aligned} \tag{7}$$

This state is labeled as HS in the main text.

Fig. 1(b)-(d) shows the behavior of D and F as a function of K_{AF}. We observe that

$$T = D + F = \frac{1}{2}\left[S_{\mathrm{tot}}^2 - S_1^2 - S_2^2 - S_3^2 - \sigma^2\right] = -\frac{1}{2} \tag{8}$$

is independent of the behavior of D and F. In the weak coupling limit, $K_{\mathrm{AF}}/t << 1$, kinetic energy gain due to fermion hopping dominates over the localized Kondo interaction term leading to a ferromagnetic (FM) core spin texture. For $K_{\mathrm{AF}} \sim 0$, we have

$$D = 3/4 \,. \tag{9}$$

Using Eq. (8), we get,

$$F = -5/4 = -1.25 \,, \tag{10}$$

for $S_{\mathrm{tot}} = (N-1)/2 = 1$. However, we find that our result $F = -1.18$ is slightly smaller than this value. With increasing values of K_{AF}, due to Kondo spin fluctuations (KSF), the spin-spin correlations decrease continuously while the spin-fermion correlations correspondingly increase such that $T = -1/2$ for $K_{\mathrm{AF}} \geq 0.5$, as shown in Fig. 1(c). Eventually, in the double

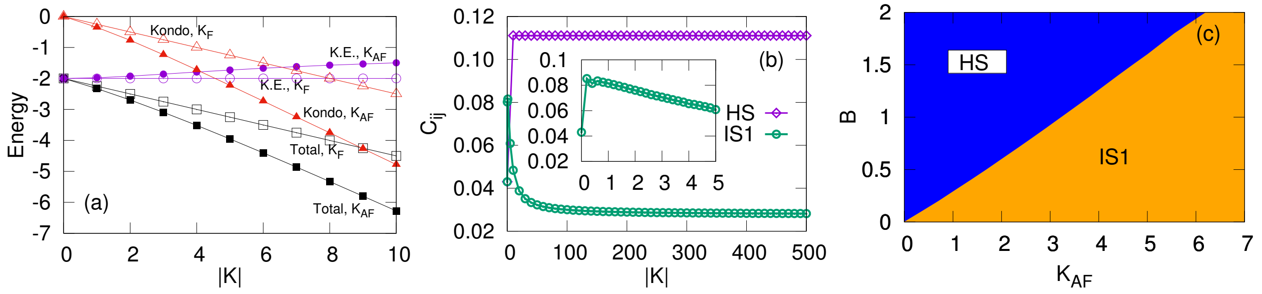

Figure 2. The ground state energy and respective contributions from the kinetic energy (KE) and Kondo interactions for K_F (open symbols) and K_AF (filled symbols). (b) Charge-charge correlations for HS and IS1 state as a function of $|K|$. The inset shows the behavior for small values of K_AF. (c) The phase diagram of electron-doped MM for $J_\mathrm{AF} = 0$.

exchange (DE) limit, $K_\mathrm{AF}/t >> 1$, the spin-spin correlation $D = 1/4$ and $F = -3/4$, implying that:

$$\begin{aligned} S^2 &= (S_1 + S_2 + S_3)^2 = \frac{11}{4} \, , \\ \text{and,} \;\; S^2_\mathrm{tot} &= (S + s)^2 = 2 \, , \end{aligned} \tag{11}$$

as expected for $S_\mathrm{tot} = (N-1)/2 = 1$.

At finite but small values of K_AF, these correlations can be modeled as,

$$\begin{aligned} D &= 3/4 + \Delta(K_\mathrm{AF}) \\ F &= -5/4 + \delta(K_\mathrm{AF}) \, , \end{aligned} \tag{12}$$

where $\Delta(K_\mathrm{AF})$ and $\delta(K_\mathrm{AF})$ represent the deviations from the respective weak-coupling limit ($K_\mathrm{AF}/t << 1$) values as a function of K_AF. For $S^2_\mathrm{tot} = 2$, it can be shown that:

$$\Delta(K_\mathrm{AF}) = \delta(K_\mathrm{AF}) \, , \tag{13}$$

implying that the effects of KSF on the spin-spin correlations is exactly compensated by the spin-fermion correlations for all values of K_AF.

Fig. 1(d) shows the longitudinal (z) and the transverse components of the spin-spin and spin-fermion correlations. While the longitudinal components exactly cancel out, the transverse components lead to the results discussed above. Thus, in this state, labeled as IS1 in the main text, the spin-spin correlations continuously decrease from $3/4$ to $1/4$ while preserving T, due to the KSF processes.

Fig. 2(a) shows the ground state energy and respective contributions from the kinetic energy (KE) and the Kondo interaction term for K_F and K_AF. For K_F, since the ground state is a fully-polarized FM, the KE contribution is independent of K_F and the Kondo contribution to the total energy $\sim K_\mathrm{F}/4$ in the strong-coupling limit. On the other hand, for K_AF, the Kondo term competes with the kinetic energy due to electron hopping in the weak Kondo-coupling limit, and in the DE limit, the Kondo contribution is proportional to $K_\mathrm{AF}/2$.

Charge-Charge Correlations

Both the phases discussed above are accompanied by different electron distributions. As the expectation value of on-site electron occupation $\langle n_i \rangle = 1/3$ (for $i = 1,2,3$) for both HS and IS1 states due to degeneracy in the ground state, we compute the charge-charge correlations (Eq. (4)) between sites i and j to ascertain the asymmetry in electron distribution in the ground state. For K_F, when HS is the ground state, it comprises of equal contributions of $\uparrow$ and $\downarrow$ spins. However, each degenerate state contains contributions from either $\uparrow$ or $\downarrow$ spin. This, in turn, would lead to non-interacting charge-charge correlations. Indeed, we find that:

$$\begin{aligned} \langle n_{i\uparrow} \rangle &= \langle n_{i\downarrow} \rangle \qquad \forall \, i \\ \text{and,} \;\; C_{ij} &= \langle n_i \rangle \langle n_j \rangle \\ &= \left(\frac{1}{3}\right)^2 = 0.11 \, , \end{aligned} \tag{14}$$

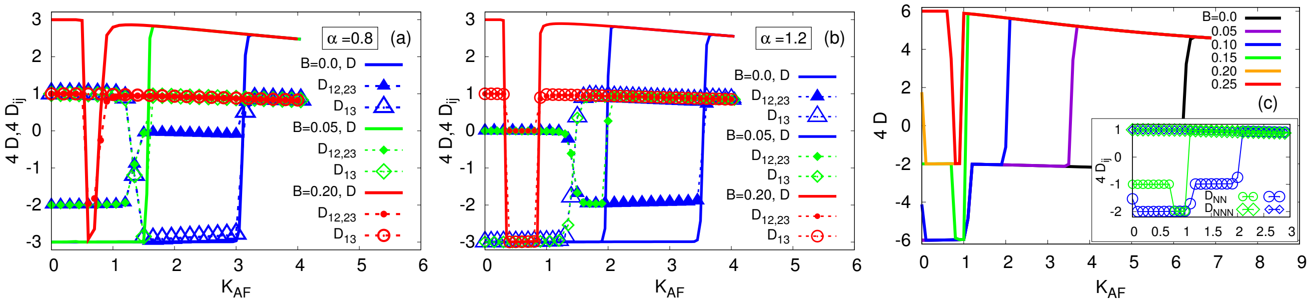

Figure 3. (a)-(b) The spin-spin correlations for asymmetric triangular MM clusters as a function of $K_{\rm AF}$ for different values of magnetic field, for: (a) $\alpha = 0.8$ and (b) $\alpha = 1.2$. (c) The spin-spin correlations for symmetric 4-site MM clusters as a function of $K_{\rm AF}$ for different values of magnetic field B, for $J_{\rm AF} = 0.2$. The inset shows the bond resolved spin-spin correlations for nearest neighbour ($D_{\rm NN}$) pairs ($ij = 12, 23, 34$ and 41), and the next nearest neighbour pairs ($D_{\rm NNN}$) ($ij = 13$ and 24).

for all $\{ij\}$ pairs, as shown in Fig 2(c). On the other hand, for $K_{\rm AF}$, when the ground state is the IS1 state, the charge-charge correlations are smaller than the corresponding non-interacting case due to Kondo spin fluctuation processes. In the DE limit, $K_{\rm AF}/t >> 1$, we find that:

$$C_{ij} \sim \left(\frac{1}{6}\right)^2 = 0.028\ , \qquad (15)$$

for all $\{ij\}$ pairs.

It is worth mentioning that, for the Kondo lattice model, with increasing values of $K_{\rm AF}$, the electron tends to form a singlet with one of the core spins. Thus, in the DE limit, the electron is localized as a magnetic polaron cloud on one of the sites. However, in the present case, finite values of charge-charge correlations suggest the absence of electron localization.

Phase Diagram In An External Magnetic Field

In the presence of an external magnetic field in the direction perpendicular to the plane of the MM, included via the Zeeman terms (Eq. (1) & (2) in the main text), KSF processes compete with the tendency of the magnetic field to drive the system towards a complete FM (HS state). The phase diagram representing this competition is shown in Fig. 2(d). At small values of $K_{\rm AF}$ and increasing values of B, the magnetic field dominates, leading to a fully polarized HS state. With increasing values of $K_{\rm AF}$, however, the Kondo spin fluctuations eventually drive the system to IS1 state, leading to spin-state switching at finite $K_{\rm AF}$. It is interesting to note the linear phase boundary.

Spin switching in SMMs – Generalization to other cases

In this section, we show that the essential results presented in this work, *viz.* multiple spin switchability in the Kondo description of doped molecular magnets, can be easily generalized to other cases. In the context of triangular MMs, of particular relevance is the asymmetry leading to an isosceles geometry. This asymmetry would affect the intramolecular exchange coupling for the core spins and the hopping parameters for the doped (itinerant) electron while the on-site Kondo coupling would remain unchanged. In general, the affect on the exchange coupling and the hopping parameters need not be same. However, for simplicity, we consider $J_{12}/t = J_{23}/t = J_{\rm AF}$; $J_{31}/t = \alpha J_{\rm AF}$ and $t_{12} = t_{23} = t$; $t_{31} = \alpha t$.

Fig. 3(a) & (b), respectively, show the behaviour of total and bond-resolved spin-spin correlations for $J_{\rm AF} = 0.2$ for $\alpha = 0.8$ and 1.2, effectively illustrating that the results for the symmetric triangular clusters can be generalized to the asymmetric cases. To begin with, variation of the total spin-spin correlations, $D = \sum_{ij} D_{ij}$, for different values of the magnetic field B is similar to the symmetric case, including a double-switching behavior in the similar range of $K_{\rm AF}$ values. However, as expected, the correlations D_{31} differs from the D_{12} and D_{23} and has been shown explicitly. Interestingly, for $\alpha > 1$, the range of $K_{\rm AF}$ values corresponding to the double-switching behaviour is greater that than for the symmetric case, implying a larger area of the re-entrant IS2 state. A systematic study and the complete phase diagram for this problem would be presented elsewhere.

Another important aspect of the model used in this work is generalization to n-clusters for $n \geq 3$. As an example, we consider a non-frustrated symmetric 4-site cluster with $J_{12}/t = J_{23}/t = J_{34}/t = J_{41}/t = J_{\rm AF}$ and $J_{13}/t = J_{24}/t = 0$. Fig. 3(c) shows the total spin-spin correlations for $J_{\rm AF} = 0.2$, for different values of magnetic field. Evidently, as a function of the Kondo coupling strength $K_{\rm AF}$ and the magnetic field B, the 4-site cluster also support multiple spin states stable over a wide range of parameters space, details of which will presented elsewhere.

References

1. Manfred Sigrist, Hirokazu Tsunetsugu and Kazuo Ueda. Rigorous results for the one-electron Kondo-lattice model. *Physical Review Letters* **67**, 2211 (1991).
